# Differential Effects of Oral Boluses of Vitamin D_2_ vs Vitamin D_3_ on Vitamin D Metabolism: A Randomized Controlled Trial

**DOI:** 10.1210/jc.2019-00207

**Published:** 2019-06-14

**Authors:** Adrian R Martineau, Kenneth E Thummel, Zhican Wang, David A Jolliffe, Barbara J Boucher, Simon J Griffin, Nita G Forouhi, Graham A Hitman

**Affiliations:** 1 Blizard Institute, Barts and The London School of Medicine and Dentistry, Queen Mary University of London, London, United Kingdom; 2 Department of Pharmaceutics, University of Washington, Seattle, Washington; 3 Medical Research Council Epidemiology Unit, University of Cambridge School of Clinical Medicine, Cambridge, United Kingdom; 4 Primary Care Unit, Department of Public Health and Primary Care, University of Cambridge School of Clinical Medicine, Cambridge, United Kingdom

## Abstract

**Context:**

Vitamin D_2_ and vitamin D_3_ have been hypothesized to exert differential effects on vitamin D metabolism.

**Objective:**

To compare the influence of administering vitamin D_2_ vs vitamin D_3_ on metabolism of vitamin D_3_.

**Methods:**

We measured baseline and 4-month serum concentrations of vitamin D_3,_ 25-hydroxyvitamin D_3_ [25(OH)D_3_], 25-hydroxyvitamin D_2_, 24R,25-dihydroxyvitamin D_3_ [24R,25(OH)_2_D_3_], 1*α*,25-dihydroxyvitamin D_3_ [1*α*,25(OH)_2_D_3_], and 4*β*,25-dihydroxyvitamin D_3_ [4*β*,25(OH)_2_D_3_] in 52 adults randomized to receive a total of four oral bolus doses of 2.5 mg vitamin D_2_ (n = 28) or vitamin D_3_ (n = 24) over four months. Metabolite-to-parent compound ratios were calculated to estimate hydroxylase activity. Pairwise before vs after comparisons were made to evaluate effects of vitamin D_2_ and vitamin D_3_ on metabolism of vitamin D. Mean postsupplementation metabolite-to-parent ratios were then compared between groups.

**Results:**

Vitamin D_2_ was less effective than vitamin D_3_ in elevating total serum 25(OH)D concentration. Vitamin D_2_ suppressed mean four-month serum concentrations of 25(OH)D_3_, 24R,25(OH)_2_D_3_, 1*α*,25(OH)_2_D_3_, and 4*β*,25(OH)_2_D_3_ and mean ratios of 25(OH)D_3_ to D_3_ and 1*α*,25(OH)_2_D_3_ to 25(OH)D_3_, while increasing the mean ratio of 24R,25(OH)_2_D_3_ to 25(OH)D_3_. Vitamin D_3_ increased mean four-month serum concentrations of 25(OH)D_3_, 24R,25(OH)_2_D_3_, 1*α*,25(OH)_2_D_3_, and 4*β*,25(OH)_2_D_3_ and the mean ratio of 24R,25(OH)_2_D_3_ to 25(OH)D_3_. Participants receiving vitamin D_2_ had lower mean postsupplementation ratios of 25(OH)D_3_ to vitamin D_3_ and 1*α*,25(OH)_2_D_3_ to 25(OH)D_3_ than those receiving vitamin D_3_. Mean postsupplementation ratios of 24R,25(OH)_2_D_3_ to 25(OH)D_3_ and 4*β*,25(OH)_2_D_3_ to 25(OH)D_3_ did not differ between groups.

**Conclusions:**

Bolus-dose vitamin D_2_ is less effective than bolus-dose vitamin D_3_ in elevating total serum 25(OH)D concentration. Administration of vitamin D_2_ reduces 25-hydroxylation of vitamin D_3_ and 1-*α* hydroxylation of 25(OH)D_3_, while increasing 24R-hydroxylation of 25(OH)D_3_.

## Teaser

Data from this randomized controlled trial show that vitamin D_2_ reduces 25-hydroxylation of vitamin D_3_ and 1-*α* hydroxylation of 25(OH)D_3_, and increases 24R-hydroxylation of 25(OH)D_3_.

**Figure 1.**
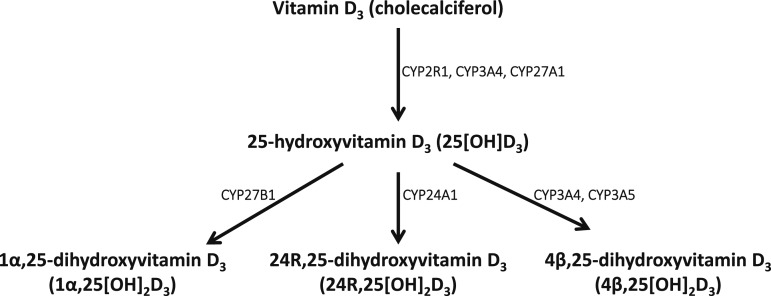
Vitamin D_3_ oxidation pathways. The monohydroxylated and dihydroxylated metabolites investigated in the current study are shown, with the cytochrome P450 enzymes catalyzing each conversion in capitals.

Vitamin D has two forms: ergocalciferol (vitamin D_2_) is synthesized *via* UV irradiation of ergosterol, a steroid found in fungi and some plants, whereas cholecalciferol (vitamin D_3_) is synthesized via UV irradiation of 7-dehydrocholesterol to previtamin D_3_, followed by a thermal isomerization step. In humans, the source of vitamin D_3_ may be endogenous (*i.e.,* obtained via cutaneous synthesis) or exogenous (*i.e.,* ingested in foods or supplements), whereas vitamin D_2_ is only available from exogenous sources. Vitamin D_2_ and vitamin D_3_ are structurally distinct: the side chain of vitamin D_2_ contains a double bond between carbons 22 and 23 and a methyl group on carbon 24, both of which are absent from the side chain of vitamin D_3_. The two forms also have differing pharmacokinetics: of 14 publications comparing effects of vitamin D_2_ vs vitamin D_3_ (1–14), all but three (12–14) reported that vitamin D_2_ was less effective than vitamin D_3_ in elevating total 25(OH)D levels. A meta-analysis of data from seven of these studies found that this effect was only statistically significant when vitamin D was administered using intermittent bolus dosing, as opposed to daily administration (15). 25-hydroxyvitamin D_3_ [25(OH)D_3_] subsequently undergoes a second hydroxylation step to form the active vitamin D metabolite 1*α*,25-dihydroxyvitamin D_3_ [1*α*,25(OH)_2_D_3_] or the inactive metabolites 24R,25-dihydroxyvitamin D_3_ [24R,25(OH)_2_D_3_] and 4*β*,25-dihydroxyvitamin D [4*β*,25(OH)_2_D_3_; Fig. 1]. It also undergoes conjugation to circulating inactive sulfate and glucuronide metabolites that may be recycled back to 25(OH)D_3_ rather than excreted (16, 17).

Administration of vitamin D_2_ has been reported to reduce circulating concentrations of 25(OH)D_3_ in eight studies (1–7, 11); a ninth study reports a nonstatistically significant trend in the same direction (12). These observations have led investigators to speculate that vitamin D_2_ may influence metabolism of vitamin D_3_. In keeping with this hypothesis, a recent study reported that administration of vitamin D_2_ increases the ratio of 24R,25(OH)_2_D_3_ to 25(OH)D_3_ in the circulation and decreases the ratio of 1*α*,25[OH]_2_D_3_ to 25(OH)D_3_, findings taken to indicate that vitamin D_2_ induces 24R-hydroxylation and suppresses 1*α*-hydroxylation of 25(OH)D_3_ (18). However, it is not yet known if these effects are vitamin D_2_-specific, because administration of vitamin D_3_ also influences the rate of conversion of parent vitamin D_3_ to its hydroxylated metabolites (19). Moreover, the influence of administering vitamin D_2_ on circulating concentrations of vitamin D_3_ and 4*β*,25(OH)_2_D_3_ has yet to be determined.

Studies making a head-to-head comparison of the influence of identical doses of vitamin D_2_ vs vitamin D_3_ on circulating concentrations of vitamin D_3_, 25(OH)D_3_ and its major dihydroxylated metabolites are needed to resolve these questions. An opportunity to conduct such an investigation recently arose in the context of a randomized controlled trial that we conducted in the United Kingdom to evaluate the effect of administration of four monthly oral doses of 2.5 mg vitamin D_2_ vs the same dose of vitamin D_3_ on glycated hemoglobin concentration among people at risk for type 2 diabetes mellitus (11). We therefore determined concentrations of vitamin D_3_, 25(OH)D_3_, 24R,25(OH)_2_D_3_, 1*α*,25(OH)_2_D_3_, and 4*β*,25(OH)_2_D_3_ in serum samples taken from a subset of trial participants before and after administration of vitamin D_2_ vs vitamin D_3_, and calculated the change in postsupplementation metabolite-to-parent ratios to gain insight into the relative effects of vitamin D_2_ vs vitamin D_3_ on the activity of enzymes catalyzing 25-hydroxylation of vitamin D_3_ and 1*α*-hydroxylation, 24R-hydroxylation, and 4*β*-hydroxylation of 25(OH)D_3_. We also measured concentrations of vitamin D_2_ and 25(OH)D_2_ in the same samples and compared the influence of vitamin D_2_ vs vitamin D_3_ on 25-hydroxylation of vitamin D_2_.

## Methods

### Trial design and participants

As previously described, we conducted a double-blind, randomized placebo-controlled trial that enrolled a total of 340 men and women aged 30 to 75 years who had been identified as being at increased risk of developing type 2 diabetes mellitus in London and Cambridge, United Kingdom (11). Full details of inclusion and exclusion criteria are described in the published protocol (20). Eligible participants were randomly allocated to one of three groups on a 1:1:1 basis within four strata defined by age (30 to 50 or 51 to 75 years) and sex, with a block size of six within each stratum. One group received four monthly oral bolus doses of 2.5 mg vitamin D_2_: each dose was presented as 5 mL Sterogyl solution (Desma Pharma, Paris, France) containing 0.5 mg vitamin D_2_ per milliliter in ethanol. The second group received four monthly oral bolus doses of 2.5 mg vitamin D_3_: each dose was presented as 5 mL Vigantol oil (Merck Serono, Darmstadt, Germany) containing 0.5 mg vitamin D_3_ per milliliter in Miglyol oil (Caesar & Loretz, Hilden, Germany). The third group received four monthly oral doses of placebo (Miglyol oil). The order of treatments within each block was determined by a computer-generated pseudo-random sequence, generated by the study medication manufacturer (Nova Laboratories, Leicester, UK). Neither the participants, the investigators, nor the laboratory staff knew the treatment allocation. Each participant was followed-up for a total of four months from their first visit; serum samples were collected at baseline and at the end of the study. Baseline and four-month serum samples taken from a subset of 28 participants and allocated to vitamin D_2_ and 25 participants allocated to vitamin D_3_ were sent for determination of concentrations of vitamin D_3_ and its metabolites as detailed below. The subset of participants contributing samples to the current study were selected on the basis that they were all recruited in London; that they had each received four directly observed doses of vitamin D_2_ or vitamin D_3_; and that they were the 28 samples in each group having the greatest volume of serum available at both baseline and four-month follow-up to be sent for further analysis. For the vitamin D_3_ group, only 25 samples had sufficient sample volume for analysis. Ethical approval for the trial was provided by the Charing Cross Medical Ethics Committee (ref 09/H0711/85) and the Cambridge Local Research Ethics Committee (ref 04/Q0108/19), and written informed consent was obtained from all participants. The trial was registered under the numbers EudraCT 2009-011264-11 and ISRCTN86515510 on 23 October 2009.

### Laboratory assays

Serum concentrations of vitamin D_3_, vitamin D_2_, 25(OH)D_3_, 25(OH)D_2_, 24R,25(OH)_2_D_3_, 1*α*,25(OH)_2_D_3_, 1*α*,25(OH)_2_D_2_, and 4*β*,25(OH)_2_D_3_ were determined by liquid chromatography-tandem mass spectrometry in the Thummel Laboratory, Department of Pharmaceutics, University of Washington, Seattle, Washington, as previously described (21). Lower limits of quantitation (LLOQ) were 0.23 nmol/L for vitamin D_3_, 0.15 nmol/L for vitamin D_2_, 0.50 nmol/L for 25(OH)D_3_, 0.24 nmol/L for 25(OH)D_2_, 0.14 nmol/L for 24R,25(OH)_2_D_3_, and 7.7 pmol/L for 1*α*,25(OH)_2_D_3_, 1*α*,25(OH)_2_D_2_ and 4*β*,25(OH)_2_D_3_. Where concentrations of a given analyte were less than the LLOQ, a value equal to the LLOQ divided by the √2 was imputed, as performed elsewhere (22). Intraday and interday coefficients of variation were <15% for all analytes, as previously reported (21).

### Sample size and statistical analyses

We estimated that paired before and after serum samples from 21 participants would need to be evaluated to have 90% power to detect a 15 nmol/L difference in 25(OH)D_3_ concentration preadministration vs postadministration of vitamin D_2_ with *α* = 0.05, based on a standard deviation for postsupplementation serum 25(OH)D_3_ concentration of 20 nmol/L (11). This sample size was inflated to 28 to allow for potential assay failure. Serum samples from a similar number of participants allocated to vitamin D_3_ were also evaluated.

Statistical analyses were conducted using GraphPad Prism version 6.04 (GraphPad Software Inc., La Jolla, CA) and STATA IC version 12 (StataCorp, College Station, TX). Intragroup differences in absolute concentrations of vitamin D_3_ and its metabolites before vs after supplementation with vitamin D_2_ or vitamin D_3_ were evaluated using paired Student *t* tests. Intergroup differences in end-study values of these parameters were evaluated with linear regression, adjusting for baseline values. Mean differences are presented with 95% CI and *P* values, with statistical significance inferred where *P* values are less than 0.05.

## Results

### Participant enrollment and baseline characteristics

**Figure 2.**
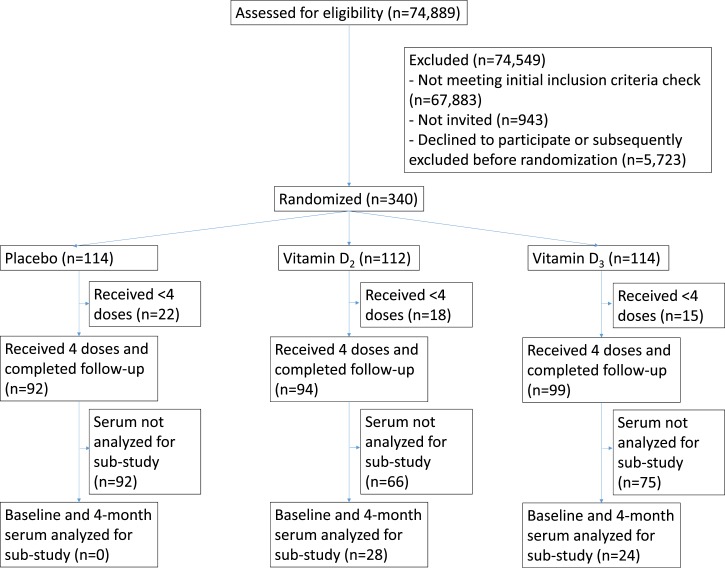
Trial profile.

A total of 340 adults were randomly assigned to receive supplementation with vitamin D_2_ (n = 112) vs vitamin D_3_ (n = 114) vs placebo (n = 114) between 2010 and 2012, of whom 285 (94 randomized to vitamin D_2_ vs 99 randomized to vitamin D_3_ vs 92 randomized to placebo) took all four doses of study medication and completed follow-up. For the current study, baseline and four-month serum samples collected from a subset of participants recruited in London who took four doses of vitamin D_2_ (n = 28) or vitamin D_3_ (n = 25) were sent for determination of concentrations of vitamin D and its metabolites (Fig. 2). Effects of the intervention on the primary outcome of the main trial, and on safety, are reported elsewhere (11). The trial ended on the date of the final study visit of the last participant to be randomized. One participant selected for the substudy and randomly assigned to vitamin D_3_ was found to have a high outlying baseline 25(OH)D_2_ concentration (51.8 nmol/L) and was excluded from statistical analyses at a reviewer’s request. Baseline characteristics of participants whose serum samples contributed to the current study are presented in Table 1. Overall, mean age was 55.6 years (SD 10.0 years) and 21 of 52 (40.4%) participants were female. Baseline demographic and clinical characteristics, serum concentrations of vitamin D_3_, vitamin D_2_ and their metabolites and metabolite-to-parent ratios were similar for those randomized to receive vitamin D_2_ vs vitamin D_3_ where measurable; concentrations of 1*α*,25(OH)_2_D_2_ were undetectable (<7.7 pmol/L) in all samples.

**Table 1.** Participants’ Baseline Characteristics by Allocation

	Characteristics	Vitamin D_2_ (n=28)	Vitamin D_3_ (n = 24)
Sex	Female, n (%)	10 (35.7)	11 (45.8)
	Male, n (%)	18 (64.3)	13 (54.2)
Mean age, y (SD)		56.0 (10.8)	55.0 (9.1)
Ethnic origin	White, n (%)	18 (64.3)	18 (75.0)
	Other, n (%)	10^*a*^ (35.7)	6 (25.0)^*b*^
Total serum concentration of vitamin D and its metabolites^*c*^	Mean total vitamin D, nmol/L (SD)^*d*^	6.5 (6.7)	8.6 (8.5)
	Mean total 25(OH)D, nmol/L (SD)^*e*^	49.7 (32.3)	45.5 (25.0)
	Mean total 25(OH)D-to-total vitamin D molar ratio (SD)	19.3 (27.5)	18.6 (22.4)
Serum concentration of vitamin D_3_ and its metabolites	Mean vitamin D_3_, nmol/L (SD)	2.9 (3.3)	3.5 (4.2)
	Mean 25(OH)D_3_, nmol/L (SD)	46.1 (32.4)	42.0 (24.7)
	Mean 1*α*,25(OH)_2_D_3_, pmol/L (SD)	77.2 (46.0)	96.9 (55.1)
	Mean 24R,25(OH)_2_D_3_, nmol/L (SD)	3.2 (2.7)	2.9 (1.8)
	Mean 4*β*,25(OH)_2_D_3_, pmol/L (SD)	126.4 (133.8)	97.1 (104.9)
	Mean 25(OH)D_3_-to-vitamin D_3_ molar ratio (SD)	39.0 (47.2)	34.1 (44.2)
	Mean 24R,25(OH)_2_D_3_-to-25(OH)D_3_ molar ratio (SD)	0.07 (0.03)	0.07 (0.04)
	Mean 1*α*,25(OH)_2_D_3_-to-25(OH)D_3_ molar ratio (SD)	0.0023 (0.0018)	0.0029 (0.0022)
	Mean 4*β*,25(OH)_2_D_3_-to-25(OH)D_3_ molar ratio (SD)	0.0024 (0.0016)	0.0020 (0.0014)
Serum concentration of vitamin D_2_ and its metabolites	Mean vitamin D_2_, nmol/L (SD)	3.6 (5.3)	5.1 (7.7)
	Mean 25(OH)D_2_, nmol/L (SD)	3.5 (1.3)	3.5 (1.5)
	Mean 1*α*,25(OH)_2_D_2_, pmol/L, (SD)	<7.7^*f*^	<7.7^*f*^
	Mean 25(OH)D_2_-to-vitamin D_2_ molar ratio (SD)	18.5 (17.2)	16.2 (19.5)

^a^ Of whom 6 were of black or black British ethnic origin and 4 were of Asian or Asian British ethnic origin.

^b^ Of whom 5 were of Asian or Asian British ethnic origin and 1 was of black or black British ethnic origin.

^c^ Not calculated for 1,25-dihydroxyvitamin D (1,25-dihydroxyvitamin D_2_ was undetectable in all) or 24,R,25-dihydroxyvitamin D/4*β*-dihydroxyvitamin D (neither 24,R,25-dihydroxyvitamin D_2_ nor 4*β*-dihydroxyvitamin D_2_ were measured).

^d^ Calculated by summing values for vitamin D_2_ and vitamin D_3._

^e^ Calculated by summing values for 25-hydroxyvitamin D_2_ and 25-hydroxyvitamin D_3._

^f^ 1*α*,25(OH)_2_D_2_ undetectable in all; lower limit of quantification for this metabolite was 7.7 pmol/L.

### Influence of vitamin D_2_ vs vitamin D_3_ on total 25(OH)D concentrations

**Figure 3.**
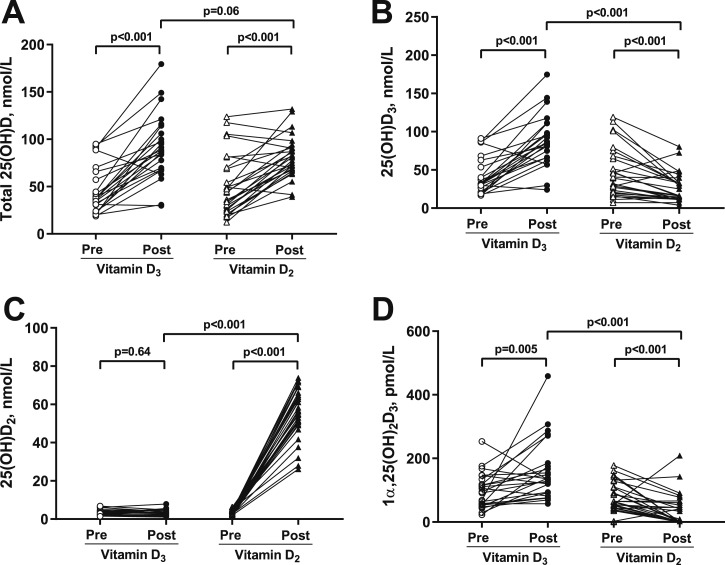
Influence of oral administration of vitamin D_3_ and vitamin D_2_ on serum concentrations of (A) total 25(OH)D, (B) 25(OH)D_3_, (C) 25(OH)D_2_, and (D) 1*α*,25(OH)_2_D_3_. Baseline and 4-mo data are presented for 24 adults receiving four bolus doses of 2.5 mg vitamin D_3_ at 0, 1, 2, and 3 mo postrandomization and 28 adults receiving an equivalent regimen of vitamin D_2_. Lines link data points from the same individual; *P* values for within-group comparisons before vs after supplementation are from paired Student *t* tests. *P* values for intergroup comparisons of postsupplementation values in participants randomized to vitamin D_3_ vs vitamin D_2_ are from linear regression with adjustment for baseline values.

Both vitamin D_2_ and vitamin D_3_ elevated total 25(OH)D concentrations at follow-up: the mean increase in total 25(OH)D concentrations after administration of vitamin D_2_ was 31.4 nmol/L (95% CI 21.5 to 41.2 nmol/L, *P* < 0.001), and the corresponding increase in total 25(OH)D concentrations after administration of vitamin D_3_ was 46.4 nmol/L (95% CI 33.7 to 59.0 nmol/L, *P* < 0.001). The difference in the mean change in total 25(OH)D concentration at follow-up for participants randomized to vitamin D_3_ vs vitamin D_2_ was 13.0 nmol/L (95% CI -0.5 to 26.6 nmol/L, *P* = 0.06; Table 2;Fig. 3). No difference in the ratio of total 25(OH)D to total parent vitamin D at follow-up was seen between participants randomized to vitamin D_2_ vs vitamin D_3_ (*P* = 0.32).

**Table 2.** Serum Concentrations of Vitamin D_3_, Vitamin D_2_ and Their Metabolites After Administration of Vitamin D_2_ vs Vitamin D_3_

	Post-Vitamin D_2_ (n = 28)	Post-Vitamin D_3_ (n = 24)	Paired Analyses				Unpaired Analysis	
			Mean Difference, Post-Vitamin vs Pre-Vitamin D_2_ (95% CI)	*P*	Mean Difference, Post-Vitamin vs Pre-Vitamin D_3_ (95% CI)	*P*	Mean Difference, Post-Vitamin D_3_ vs Post-Vitamin D_2_ (95% CI)^*a*^	*P*
Total vitamin D and its metabolites^*b*^								
Mean total vitamin D, nmol/L (SD)^*c*^	7.4 (7.8)	6.8 (5.7)	0.9 (−3.1 to 5.0)	0.65	−1.7 (−5.8 to 2.3)	0.39	−0.6 (−4.6 to 3.3)	0.75
Mean total 25(OH)D, nmol/L (SD) ^*d*^	81.0 (21.9)	91.9 (34.5)	31.4 (21.5 to 41.2)	<0.001	46.4 (33.7 to 59.0)	<0.001	13.0 (−0.5 to 26.6)	0.06
Mean total 25(OH)D-to-total vitamin D molar ratio (SD)	27.8 (49.1)	17.6 (7.9)	8.5 (−13.9 to 31.0)	0.44	−1.1 (−10.2 to 8.1)	0.81	−10.3 (−30.9 to 10.3)	0.32
Vitamin D_3_ and its metabolites								
Mean vitamin D_3_, nmol/L (SD)	5.6 (7.3)	6.3 (5.5)	2.7 (−0.3 to 5.8)	0.07	2.8 (0.3 to 5.3)	0.03	0.5 (−3.2 to 4.1)	0.80
Mean 25(OH)D_3_, nmol/L (SD)	26.2 (19.1)	88.2 (34.1)	−19.9 (−29.3 to −10.5)	<0.001	46.2 (33.3 to 59.2)	<0.001	64.0 (51.0 to 77.1)	<0.001
Mean 24R,25(OH)_2_D_3_, nmol/L	2.0 (1.5)	7.7 (3.0)	−1.2 (−2.0 to −0.3)	0.007	4.8 (3.7 to 6.0)	<0.001	5.8 (4.7 to 7.0)	<0.001
Mean 1*α*,25(OH)_2_D_3_, pmol/L	36.0 (50.5)	161.4 (95.4)	−41.1 (−63.8 to 18.5)	<0.001	64.5 (21.4 to 107.6)	0.005	119.7 (77.6 to 161.8)	<0.001
Mean 4*β*,25(OH)_2_D_3_, pmol/L	73.2 (81.9)	305.4 (438.6)	−53.2 (−99.2 to −7.2)	0.03	208.3 (40.7 to 375.9)	0.02	258.8 (98.0 to 419.7)	0.002
Mean 25(OH)D_3_-to-vitamin D_3_ molar ratio (SD)	9.3 (11.4)	18.2 (7.7)	−29.7 (−48.5 to −10.9)	0.003	−15.9 (−34.5 to 2.7)	0.09	9.0 (3.4 to 14.6)	0.002
Mean 24R,25(OH)_2_D_3_-to-25(OH)D_3_ molar ratio (SD)	0.0822 (0.0435)	0.0928 (0.0297)	0.0163 (0.0010 to 0.0315)	0.04	0.0214 (0.0069 to 0.0360)	0.006	0.0078 (−0.0114 to 0.0270)	0.42
Mean 1*α*,25(OH)_2_D_3_-to-25(OH)D_3_ molar ratio (SD)	0.0011 (0.0015)	0.0020 (0.0011)	−0.0012 (−0.0022 to −0.0002)	0.02	−0.0009 (−0.0020 to 0.0001)	0.06	0.0009 (0.0001 to 0.0017)	0.03
Mean 4*β*,25(OH)_2_D_3_-to-25(OH)D_3_ molar ratio (SD)	0.0024 (0.0022)	0.0030 (0.0027)	0.0000 (−0.0009 to 0.0009)	0.97	0.0010 (−0.0001 to 0.0021)	0.06	0.0008 (−0.0006 to 0.0021)	0.25
Vitamin D_2_ and its metabolites								
Mean vitamin D_2_, nmol/L (SD)	1.8 (1.1)	0.6 (0.8)	−1.8 (−4.0 to 0.3)	0.09	−4.5 (−7.7 to −1.3)	0.008	−1.2 (−1.8 to −0.6)	<0.001
Mean 25(OH)D_2_, nmol/L (SD)	54.8 (12.9)	3.6 (1.5)	51.2 (46.2 to 56.3)	<0.001	0.1 (−0.5–0.7)	0.64	−51.1 (-56.5 to −45.7)	<0.001
Mean 1*α*,25(OH)_2_D_2_, pmol/L	<7.7^*e*^	<7.7^*e*^	—	—		—	—	—
Mean 25(OH)D_2_-to-vitamin D_2_ molar ratio, (SD)	111.0 (185.2)	24.6 (18.5)	92.5 (21.8 to 163.3)	0.01	8.4 (−0.8 to 17.7)	0.07	−83.7 (−160.0 to −7.4)	0.03

^a^ Adjusted for baseline value.

^b^ Not calculated for 1,25-dihydroxyvitamin D (1,25-dihydroxyvitamin D_2_ was undetectable in all) or 24,R,25-dihydroxyvitamin D/4*β*-dihydroxyvitamin D (neither 24,R,25-dihydroxyvitamin D_2_ nor 4*β*-dihydroxyvitamin D_2_ were measured).

^c^ Calculated by summing values for vitamin D_2_ and vitamin D_3_.

^d^ Calculated by summing values for 25-hydroxyvitamin D_2_ and 25-hydroxyvitamin D_3_.

^e^ 1*α*,25(OH)_2_D_2_ undetectable in all; LLOQ for this metabolite was 7.7 pmol/L.

### Influence of vitamin D_2_ on metabolism of vitamin D_3_

To characterize effects of vitamin D_2_ on metabolism of D_3_, we conducted pairwise statistical analyses comparing circulating concentrations of vitamin D_3_ and its metabolites in 28 individuals before vs after oral administration of four monthly doses of 2.5 mg vitamin D_2_. Results are presented in Table 2. Administration of vitamin D_2_ had no statistically significant effect on serum concentrations of vitamin D_3_ (*P* = 0.07), but it did reduce mean serum concentrations of 25(OH)D_3_ (43.2% decrease, *P* < 0.001; Fig. 3), 24R,25(OH)_2_D_3_ (37.5% decrease, *P* = 0.007), 1*α*,25(OH)_2_D_3_ (53.4% decrease, *P* < 0.001; Fig. 3) and 4*β*,25(OH)_2_D_3_ (42.0% decrease, *P* = 0.03). Administration of vitamin D_2_ reduced molar ratios of 25(OH)D_3_ to vitamin D_3_ (76.2% decrease, *P* = 0.003) and 1*α*,25(OH)_2_D_3_ to 25(OH)D_3_ (52.2% decrease, *P* = 0.02), but increased the molar ratio of 24R,25(OH)_2_D_3_ to 25(OH)D_3_ (24.5% increase, *P* = 0.04). No statistically significant effect of vitamin D_2_ on the molar ratio of 4*β*,25(OH)_2_D_3_ to 25(OH)D_3_ was seen (*P* = 0.97).

### Influence of vitamin D_3_ on its own metabolism

Having characterized the effects of vitamin D_2_ on metabolism of vitamin D_3_, we proceeded to conduct a second set of pairwise before and after statistical analyses to evaluate the effects of vitamin D_3_ on the same biochemical parameters in a separate group of 24 individuals who received four monthly doses of 2.5 mg vitamin D_3_. Results of these analyses (Table 2) show that administration of vitamin D_3_ elevated serum concentrations of vitamin D_3_ (80.0% increase, *P* = 0.03), 25(OH)D_3_ (110.0% increase, *P* < 0.001; Fig. 3), 24R,25(OH)_2_D_3_ (165.5% increase, *P* < 0.001), 1*α*,25(OH)_2_D_3_ (66.6% increase, *P* = 0.005), and 4*β*,25(OH)_2_D_3_ (214.5% increase, *P* = 0.02). Administration of vitamin D_3_ also increased the ratio of 24R,25(OH)_2_D_3_ to 25(OH)D_3_ (32.6% increase, *P* = 0.006) but had no statistically significant effect on ratios of 25(OH)D_3_ to vitamin D_3_ (*P* = 0.09), 1*α*,25(OH)_2_D_3_ to 25(OH)D_3_ (*P* = 0.06), or 4*β*,25(OH)_2_D_3_ to 25(OH)D_3_ (*P* = 0.06).

### Comparing effects of vitamin D_2_ vs vitamin D_3_ on metabolism of vitamin D_3_

To determine whether the two forms of vitamin D exerted different effects on metabolism of vitamin D_3_, we undertook unpaired statistical analyses comparing postsupplementation values of vitamin D_3_ metabolite-to-parent ratios between individuals supplemented with vitamin D_2_ vs vitamin D_3_, using linear regression with adjustment for baseline values. Results of these analyses (Table 2) reveal that participants receiving vitamin D_2_ had lower mean postsupplementation ratios of 25(OH)D_3_ to vitamin D_3_ (*P* = 0.002) and 1*α*,25(OH)2D_3_ to 25(OH)D_3_ (*P* = 0.03) than those who received vitamin D_3_. No statistically significant difference in mean postsupplementation ratios of 24R,25(OH)_2_D_3_ to 25(OH)D_3_ (*P* = 0.42) or 4*β*,25(OH)_2_D_3_ to 25(OH)D_3_ (*P* = 0.25) was seen between participants receiving vitamin D_2_ vs vitamin D_3_.

### Influence of vitamin D_3_ vs vitamin D_2_ on metabolism of vitamin D_2_

Although the primary focus of our study was to compare the effects of vitamin D_2_ vs vitamin D_3_ on metabolism of vitamin D_3_, limited data were also available to evaluate relative effects of the two forms of vitamin D on metabolism of vitamin D_2_ (Table 2). Pairwise analyses revealed that administration of vitamin D_2_ increased mean serum 25(OH)D_2_ concentration (*P* < 0.001; Fig. 3) and mean 25(OH)D_2_-to-D_2_ molar ratio (*P* = 0.01), but had no statistically significant effect on mean serum concentration of vitamin D_2_ itself (*P* = 0.09). By contrast, administration of vitamin D_3_ reduced vitamin D_2_ concentrations over time (*P* = 0.008) but did not influence 25(OH)D_2_ concentrations (*P* = 0.64; Fig. 3) or 25(OH)D_2_-to-D_2_ ratio (*P* = 0.07). Unpaired analysis comparing postsupplementation 25(OH)D_2_-to-D_2_ ratios between individuals supplemented with vitamin D_2_ vs vitamin D_3_, with adjustment for baseline values, showed that the mean postsupplementation ratio of 25(OH)D_2_ to vitamin D_2_ among participants receiving vitamin D_2_ was higher than that of participants who received vitamin D_3_ (*P* = 0.03).

## Discussion

To our knowledge, this is the first investigation to evaluate the influence of vitamin D_2_ on circulating concentrations of parent vitamin D_3_ and its dihydroxylated metabolite 4*β*,25(OH)_2_D_3_ in addition to serum concentrations of 25(OH)D_2_ and 25(OH)D_3_. We found that administration of vitamin D_2_ exerted a greater inhibitory effect than administration of vitamin D_3_ on mean ratios of 25(OH)D_3_ to D_3_ and 1*α*,25(OH)_2_D_3_ to 25(OH)D_3_ in the circulation. We also observed that vitamin D_2_ and vitamin D_3_ increased the mean 24R,25(OH)_2_D_3_-to-25(OH)D_3_ ratio to a similar extent, and that neither form of vitamin D had a statistically significant effect on the mean serum 4*β*,25(OH)_2_D_3_-to-25(OH)D_3_ ratio. By contrast with findings of a recently published study (6), we found that administration of vitamin D_3_ did not suppress serum concentrations of 25(OH)D_2_, nor did it influence mean serum 25(OH)D_2_-to-vitamin D_2_ ratio. Administration of vitamin D_2_ resulted in an increase in the mean 25(OH)D_2_-to-D_2_ ratio.

Our findings are consistent with reports that administration of vitamin D_2_ reduces serum concentrations of 25(OH)D_3_ (1–4), and that this phenomenon is associated with an increase in the ratio of 24R,25(OH)_2_D_3_ to 25(OH)D_3_ and a decrease in the ratio of 1*α*,25(OH)2D_3_ to 25(OH)D_3_ in the circulation. Interestingly, serum 25(OH)D_2_ concentrations were markedly elevated in all study participants receiving vitamin D_2_, consistent with induction of the vitamin D_2_ 25-hydroxylation pathway, although there was an opposite effect on 25(OH)D_3_ and rate of formation in the same treated individuals (Fig. 3), raising the possibility that different enzymes might catalyze 25-hydroxylation of the two forms of vitamin D. Alternatively, the decline of 25(OH)D_3_ following administration of vitamin D_2_ may reflect competition of vitamin D_2_ for the same 25-hydroxylation pathway as vitamin D_3_. Although changes in metabolite-to-parent ratios may reflect alteration in rates of conversion of one metabolite to another, they could also be explained by removal of vitamin D and its metabolites from the circulation (*e.g.,* via direct excretion or disposition into depots such as adipose tissue and muscle). Further investigations to compare the effects of different forms of vitamin D on expression and activity of the enzymes responsible for metabolizing vitamin D_3_ are needed to resolve the question of whether changes in metabolite-to-parent ratios truly reflect changes in activity of cytochrome P450 enzymes. Such studies would potentially require liver and renal biopsies: both are invasive procedures and their inclusion in a study protocol could raise issues relating to ethics and acceptability to participants.

One aspect in which our findings differ from those of other investigators relates to the lack of detectable 1,25-dihydroxyvitamin D_2_ at follow-up among participants receiving vitamin D_2_ in our study. By contrast, Biancuzzo *et al.* reported that daily administration of 1,000 IU vitamin D_2_ for 11 weeks induced a mean increase in serum 1,25(OH)_2_D_2_ concentration of 5.2 pg/mL (13.5 pmol/L) (14). This difference may reflect use of intermittent bolus dosing in the current study, which contrasts with the daily dosing regimen used by Biancuzzo *et al.*

Our findings provide insights into the differential effects of ergocalciferol and cholecalciferol on vitamin D metabolism, however, from the clinician’s perspective, a key question relates to relative effects of the two forms of vitamin D on total 25(OH)D levels, which reflect vitamin D status. Among substudy participants, the mean increase in total 25(OH)D for participants randomized to vitamin D_2_ (n = 28) vs vitamin D_3_ (n = 25) was 31.4 nmol/L vs 46.4 nmol/L, respectively (*P* for intergroup comparison = 0.06). This trend is in keeping with findings from the main trial, in which the difference in increase in total 25(OH)D for participants randomized to vitamin D_2_ (n = 112) vs D_3_ (n= 114; 31.2 vs 38.3 nmol/L increase, respectively) attained statistical significance (*P* = 0.03).

Our study has several strengths. Participants randomized to vitamin D_2_ vs D_3_ were well matched with regard to baseline characteristics, and directly observed administration of vitamin D_2_ and vitamin D_3_ at identical doses via the same route allowed for a head-to-head comparison of their effects. Determination of concentrations of parent vitamin D_2_ and D_3_, 25(OH)D_2_, 25(OH)D_3_, and its major dihydroxylated metabolites allowed us to compare effects of vitamin D_2_ vs vitamin D_3_ on both the synthesis and the catabolism of 25(OH)D_3_. Moreover, we utilized the gold standard method (liquid chromatography-tandem mass spectrometry) to measure concentrations of vitamin D and its metabolites with high degrees of accuracy and sensitivity, avoiding issues of cross-reactivity between metabolites of vitamin D_2_ and vitamin D_3_ that may arise with immunoassays (23).

Our study also has some limitations. We measured concentrations of vitamin D metabolites at a single time point, one month after the fourth bolus dose was given; thus, we do not capture the pharmacokinetics of vitamin D metabolism at multiple points over the period of the dosing interval. In particular, conclusions relating to concentrations of parent vitamin D_2_ and vitamin D_3_ in the circulation should be guarded, because of their short half-life. Participants all had an elevated risk of type 2 diabetes mellitus: thus, our findings cannot necessarily be generalized to other groups. However, we have no specific reason to believe that effects of vitamin D_2_ are likely to be different in this group compared with the general population. We did not measure concentrations of 1,24,25-trihydroxyvitamin D_3_ or 24R,25(OH)_2_D_2_: this could have provided insights into the effects of vitamin D_2_ vs vitamin D_3_ on 24-hydroxylation of 1*α*,25(OH)_2_D_3_ and 25(OH)D_2_, respectively. Preparations of vitamin D_2_ and vitamin D_3_ were presented in different vehicles (alcohol vs oily solution, respectively), which could theoretically have impacted differently on absorption and/or metabolism. However, a study in schoolchildren comparing ethanol vs oil as a vehicle for a weekly oral dose of 14,000 IU vitamin D_3_ showed no difference in the 25(OH)D response to supplementation between groups over eight weeks (24), rendering this explanation for the findings in the current study unlikely.

In conclusion, the current study confirms reports that vitamin D_2_ is less effective than vitamin D_3_ in elevating total 25(OH)D levels, and extends prior findings by showing that administration of vitamin D_2_ reduces 25-hydroxylation of vitamin D_3_ and 1-*α* hydroxylation of 25(OH)D_3_ and increases 24R-hydroxylation of 25(OH)D_3_.

## Abbreviations:

1*α*,25(OH)_2_D_3_: 1*α*,25-dihydroxyvitamin D_3_
4*β*,25(OH)_2_D_3_: 4*β*,25-dihydroxyvitamin D_3_
24R,25(OH)_2_D_3_: 24R,25-dihydroxyvitamin D_3_
25(OH)D: 25-hydroxyvitamin D
25(OH)D_3_: 25-hydroxyvitamin D_3_
LLOQ: lower limit of quantitation
